# Remodeling tumor microenvironment using pH-sensitive biomimetic co-delivery of TRAIL/R848 liposomes against colorectal cancer

**DOI:** 10.32604/or.2024.045564

**Published:** 2024-10-16

**Authors:** YONGJIAN HUANG, JINZHOU WANG, JIUHUA XU, NING RUAN

**Affiliations:** Department of Gastrointestinal Surgery, The First Affiliated Hospital of Fujian Medical University, Fuzhou, 350005, China

**Keywords:** Colorectal cancer, Plasmid TRAIL (pTRAIL), R848, Tumor-associated macrophages

## Abstract

**Background:**

Despite significant advancements in the development of anticancer therapies over the past few decades, the clinical management of colorectal cancer remains a challenging task. This study aims to investigate the inhibitory effects of cancer-targeting liposomes against colorectal cancer.

**Materials and Methods:**

Liposomes consisting of 3β-[N-(N′, N′-dimethylamino ethane)carbamoyl]-cholesterol (DC-CHOL), cholesterol (CHOL), and dioleoylphosphatidylethanolamine (DOPE) at a molar ratio of 1:1:0.5 were created and used as carriers to deliver an apoptosis-inducing plasmid encoding the tumor necrosis factor-related apoptosis-inducing ligand (pTRAIL) gene, along with the toll-like receptor (TLR7) agonist Rsiquimod (R848). The rationale behind this design is that pTRAIL can trigger cancer cell apoptosis by activating the DR4/5 receptor, while R848 can stimulate the immune microenvironment.

**Results:**

Experimental results demonstrated the synergistic effects of R848 and pTRAIL encapsulated by liposomes (RTL) in suppressing the proliferation of colorectal cancer cells. Moreover, further *in vivo* investigations revealed the strong anti-tumor efficacy of RTL in xenograft and orthotropic *in situ* models of colorectal cancer.

**Conclusions:**

These findings collectively highlight the therapeutic potential of R848/pTRAIL-loaded liposomes in the treatment of colorectal cancer.

## Introduction

Global cancer statistics indicate that colorectal cancer has become one of the leading malignancies with an increasing rate of mortality [1,2]. In addition to conventional therapeutic interventions, immunotherapy has emerged as one of the promising strategies that is revolutionizing the field of cancer treatment [3,4]. The application of immune checkpoint inhibitors (ICIs) has shown clinical benefit in improving the survival of patients with colorectal cancer [5]. Nevertheless, a proportion of cancer patients fail to respond to immunotherapy, which can be partially attributed to the immunosuppression in the tumor microenvironment (TME) [6,7]. Therefore, how to stimulate TME-related immunity is a central topic in augmenting the efficacy of immunotherapy.

The tumor necrosis factor-related apoptosis-inducing ligand (TRAIL) can act as an apoptosis-inducing factor to trigger programmed cell death in cancer cells by binding to the death receptors (DR4 and DR5) [4,8]. The potential anticancer efficacy of recombinant human TRAIL protein (Rh-TRAIL) has been investigated in clinical trials [9]. Nevertheless, the poor pharmacokinetic characteristics of Rh-TRAIL limited its clinical treatment outcome [10]. Without proper conjugation, TRAIL protein can be degraded and cleared rapidly by the kidney [11]. It was reported that TRAIL has a short half-life of 3–5 min after being administrated into the rodents [12]. The half-life of TRAIL protein is longer in non-human primates but is still unsatisfactory to achieve a sustained anticancer dose [13]. An alternative strategy is to deliver TRAIL-encoding plasmid (pTRAIL) to tumor tissues for continuous expression of TRAIL protein. The delivery of plasmid DNA can also exert an immunogenic effect in the TME [14]. Liposome formulation is a widely used approach to encapsulate large-sized DNA molecules and offer substantial protection against degradation [15].

Rsiquimod (R848), a toll**-**like receptor 7 (TLR7) agonist, has been demonstrated with potent anti-cancer effects in different animal models of cancer [16]. In the TME, R484 can induce the anti-tumorigenic polarization of macrophages [17]. Systemic administration of R848 significantly activates TLR7-dependent signaling cascades in different immune cells, such as dendritic cells (DCs) and macrophages [18]. However, free R848 also exhibits poor pharmacokinetic characteristics in blood circulation. Over the past few decades, liposomes have emerged as the most viable drug formulation for targeted delivery *in vivo*, offering high delivery efficacy and safety. Importantly, the composition of liposomes can be tailored by combining different phospholipids with other molecules to achieve preferential delivery to tumor tissues [19].

In the present study, we aim to develop a liposomal formulation that can co-deliver pTRAIL and the TLR7 agonist R848 to simultaneously induce cancer cell death and stimulate the immune microenvironment in an animal model of colorectal tumors. R848-encapsulated (R848 Lipo) and pTRAIL-encapsulated liposomes (pTRAIL Lipo) were prepared to compare their anticancer effects with the one containing both R848 and pTRAIL (RTL). We investigated the anticancer effects of these liposomal formulations in both the xenograft tumor model and the orthotropic *in situ* model of colorectal cancer. Our results demonstrated the potent tumoricidal effect and immunomodulatory effect of RTL in the mouse models of colorectal cancer.

## Materials and Methods

### Liposome preparation

1,2-Dioleoyl-3-phosphatidylethanolamine (DOPE) was purchased from Avanti Polar Lipids (Alabaster, USA); 3β-[N-(N′,N′-dimethylaminoethane)-carbamoyl] cholesterol (DC-CHOL), cholesterol (CHOL), dimethyl sulfoxide (DMSO), and dichloromethane (DCM) were obtained from Sigma Aldrich (St. Louis, USA). The lipid precursors DC-CHOL, CHOL and DOPE were mixed at a molar ratio of 1:1:0.5 after the dose optimization for liposome formation. DC-CHOL, CHOL and DOPE were initially dissolved in DCM as the organic phase, which was then evaporated at 45°C using a vacuum rotary evaporator (Buchi R 200/205, Tokyo, Japan). The dried organic phase was re-constituted in methanol, and lyophilized using sucrose as the cryoprotectant [20].

### R848 encapsulation

TLR7 agonist R848 was purchased from Sigma Aldrich (St. Louis, USA) and reconstituted in a methanol solution to create the aqueous phase. To generate R848 liposome, DC-CHOL, CHOL and DOPE were initially dissolved in DCM as the organic phase and then evaporated under vacuum. The dried lipids were dissolved in the R848-containing aqueous phase. The resultant mixture of R848 and lipids (RLs) was sonicated at room temperature for 5 min. Afterward, the solution was centrifuged at 1000 × g for 15 min to remove any unincorporated drugs and the liposomes were collected in the supernatant. For small volume preparations, the suspension was dispersed and filtered through a polycarbonate membrane with a pore size of ~200 nm using an extruder (Avanti Polar Lipids, Alabaster, USA). For large amount preparations, liposomes were homogenized in a Lipex® Extruder device (Northern Lipids Inc., Burnaby, Canada) under nitrogen pressure. The concentration of R848 encapsulated in the liposomal vehicles was determined using a spectrophotometer (Nanodrop, Thermo Fischer Scientific, Waltham, USA) at an average diameter of 320 nm [21]. The prepared R848 liposomes were lyophilized and stored at 4°C.

### pDNA encapsulation and R848/pTRAIL liposome (RTL) generation

Plasmid DNA (pDNA) containing luciferase reporter gene, GFP reporter or pTRAIL was initially produced in *Escherichia coli* in 2 L of LB medium containing antibiotics (kanamycin). Plasmid extraction was performed using the Endotoxin-free Plasmid DNA Purification Kit (Macherey-Nager, Landsmeer, Netherlands) according to the manufacturer’s instructions. The concentration of purified pDNA was determined by spectrophotometry at 260 nm (Nanodrop, Thermo Fischer Scientific, Waltham USA). The extracted pDNA was re-constituted in sterile PBS. To generate pDNA liposomes, the dried lipid precursors were hydrated in PBS-pDNA solution and sonicated at room temperature for 5 min. To generate R848/pTRAIL liposome (RTL), the R848 liposome and pTRAIL liposome were mixed at a weight ratio of 1:1. The resulting liposome formulations were lyophilized using sucrose as the cryoprotectant and stored at 4°C before usage.

### Characterization of liposomes

R848 concentration in the liposomal sample was determined using a UV–Vis spectrophotometer at a wavelength of 320 nm [22]. The encapsulation efficiency (EE%) of the liposomes was calculated as the ratio of the amount of G848 encapsulated in the liposomes to the total drug amount used for preparation. The zeta potential and average particle size of R848 liposome, pDNA liposome, and R848/pDNA liposome were measured through dynamic light scattering (DLS) using the Zetasizer Nano S instrument (Malvern Panalytical Instruments, Ltd., Malvern, UK). For zeta potential measurement, an aqueous sample was loaded into the capillary cell with two conductive electrodes connected with the instrument’s applied voltage. The 632 nm HeNe laser was operated at a 173 degree detector angle during the measurement, and the zeta potential was detected using the built-in software. The surface morphology of the liposomes was characterized by scanning electron microscopy (SEM) (JSM-IT210, JEOL, Tokyo, Japan) at 100 kV, with an accelerating voltage of 5 kV and a deceleration voltage of 2.5 kV.

The long-term serum stability of the liposomal complexes was assessed by measuring particle size changes in FBS (10%)-supplemented phosphate-buffered saline (PBS) at 4°C or 37°C, using the Zetasizer Nano S dynamic light scatter. For the *in vitro* release kinetics determination, R848 liposome samples in water were dialyzed in 50 mL of PBS at 37°C using SnakeSkin® dialysis tubing (molecular weight cut-off, MWCO of 1.4 kDa). At predetermined time points, 1 mL of the PBS solution was removed for R8484 concentration determination, and an equal volume of fresh PBS was replenished to maintain sink conditions [23].

### Cell culture

The murine CT26 colorectal cancer cell line, human HCT116 colorectal cancer cell line, and THP-1 human monocyte cell line were acquired from the American Type Culture Collection (ATCC, Rockville, Maryland, USA). All cells were short tandem repeat (STR) profiled by the supplier, and underwent routine mycoplasma testing to eliminate mycoplasma contamination. The cells were cultivated in Dulbecco’s modified Eagle’s medium **(**DMEM, Gibco, Grand Island, USA) supplemented with 10% fetal bovine serum (FBS, Gibco) and antibiotics (100 μg mL^−1^ of streptomycin and 100 U mL^−1^ of penicillin) at 37°C and 5% CO_2_. THP-1 cells were cultivated in Roswell Park Memorial Institute (RPMI)-1640 medium (Gibco) with the same supplements. The M2 polarization of THP-1 cells was conducted based on the protocol of a previous study [24]. M2-polarized THP-1 cells were then co-cultured with HCT116 cells in a 12-well transwell chamber cassette (Corning, NY, USA) for 48 h to mimic the impact of tumor-associated macrophages on colon cancer cells.

### Verification of pDNA delivery by liposome

Colorectal cancer cells were seeded in 24-well plates at a density of 2 × 10^5^ cells/well. The culture medium was removed at the time of transfection, and the cells were washed twice with PBS. PEI2.5K Transfection Reagent (Maokangbio, Shanghai, China) was used as a positive control to transfect plasmid DNA into the cells (1.6 μg pDNA per well) following the supplier’s instructions. For the delivery of encapsulated pDNA, cells were incubated with pDNA liposome (1.6 μg pDNA per well) in serum-free DMEM. After 24 h incubation, the medium was replaced with fresh DMEM supplemented with 10% FBS, and the cells were further cultured for another 48 h at 37°C. Subsequently, the efficiency of liposome uptake or transfection was determined using a luciferase assay kit (Beyotime Biotechnology, Beijing, China) based on the manufacturer’s instructions.

### Cell viability assay

Cell viability was determined using a CCK-8 assay kit (Beyotime Biotechnology, Beijing, China) according to a published method [25]. Cells were inoculated in a 96-well plate at a density of 1 × 10^4^ per well. After reaching 70% confluency, the cells were incubated with R848 liposome complexes or free R848 at a series of diluted concentrations for 48 h. Afterward, 10 μL of CCK8 reagent (Solarbio, Beijing, China) was added into each well for 1-h incubation at 37°C. The light absorption value (OD value) in each well was detected at 450 nm wavelength using a Synergy H1 microplate reader (Winooski, Vermont, USA). The cell viability was also confirmed using a Calcein/PI Cell Activity and Cytotoxicity Test Kit (YaJi Biological, Shanghai, China).

### RT-qPCR analysis

Total RNA was extracted from cultured cells using TRIpure Total RNA Extraction Reagent (ELK Biotechnology, Wuhan, China) according to the manufacturer’s instructions. The cDNA was synthesized from 2 μg RNA samples using M-MLV Reverse Transcriptase Kit (ELK Biotechnology). qPCR analysis of the cDNA was conducted using QuFast SYBR Green PCR Master Mix (ELK Biotechnology) on the StepOne™ Real-Time PCR System (Life technologies, CA, USA). Relative gene expression was calculated by the 2^−∆∆CT^ method, with β-Actin as the endogenous control. The primer sequences are provided below:

β-Actin: F-GTCCACCGCAAATGCTTCTA; R-TGCTGTCACCTTCACCGTTC.

E-Cadherin: F-CGAGAGCTACACGTTCACGG; R-GGGTGTCGAGGGAAAAATAGG.

N-Cadherin: F-AGCCAACCTTAACTGAGGAGT; R- GGCAAGTTGATTGGAGGGATG.

Vimentin: F-GACGCCATCAACACCGAGTT; R-CTTTGTCGTTGGTTAGCTGGT.

Snail: F-TCGGAAGCCTAACTACAGCGA; R-AGATGAGCATTGGCAGCGAG.

Slug: F-CGAACTGGACACACATACAGTG; R-CTGAGGATCTCTGGTTGTGGT.

Twist: F-GTCCGCAGTCTTACGAGGAG; R-GCTTGAGGGTCTGAATCTTGCT.

### In vivo investigations

The BALB/c wildtype mice (5-week-old, female) and BALB/c nude mice (6-week-old, female) were obtained from the Shanghai Laboratory Animal Center, Chinese Academy of Sciences (CAS, Shanghai, China). The animals were housed in a pathogen-free (SPF) facility with a 12-h light/dark cycle and provided with free access to food and water. All animal experimental protocols were approved by the Institutional Animal Care and Use Committee (IACUC) of the First Affiliated Hospital of Fujian Medical University, and the experimental procedures complied with the Animal Management Rules of the Ministry of Health of the People’s Republic of China.

BALB/c nude mice were used to establish the subcutaneous xenograft model, while BALB/c wildtype mice were employed for the orthotropic *in situ* models of colorectal cancer. In the xenograft model, 1 × 10^6^ HCT116 human colon cancer cells in 0.1 ml saline were injected into the right flank of each mouse. In the orthotropic *in situ* model, 5 × 10^5^ CT26 cells in 0.1 ml saline were inoculated into the colon tissue. When the tumor size reached about 100 mm^3^, the animals were randomly assigned to different groups: control group (empty liposome); R848 liposome; pTRAIL liposome; and RTL liposome (n = 5 animals in each group of the xenograft model; n = 10 animals in each group of the orthotropic *in situ* model). The intravenous injection of liposome sample was conducted every five days at a dosage of 10 mg/kg for each formulation via the tail vein. Tumor volume was measured every three days, using the following formula: Tumor volume (V, mm^3^) = (long diameter) × (short diameter)^2^/2. After a total of 5 doses, the mice were sacrificed by cervical dislocation and the tumor samples were harvested for further analysis. Cell death events in the tumor samples were analyzed by a TdT-mediated dUTP nick end labeling (TUNEL) staining kit (*In Situ* Cell Death Detection Kit, Fluorescein, Sigma, Darmstadt, Germany).

### Cytokine detection

To detect the serum levels of cytokines, 2 mL of plasma was extracted from the vein blood by centrifuging the samples at 1000 × g for 10 min. Heparin was used during blood sample collection to prevent coagulation. The relative levels of cytokines (Transforming growth factor beta (TGF-β), macrophage migration inhibitory factor (MIF) and interferon gamma (IFN-γ)) were determined using commercial ELISA kits (Sigma, Darmstadt, Germany). The concentration of each cytokine was determined through linear regression analysis of the standards.

### Statistical analysis

All data were expressed as the mean ± standard deviation (S.D.) from at least three experiments or samples. Statistical analysis was performed using GraphPad Prism 6 software (GraphPad software, New York, USA). The unpaired Student’s *t*-test was used to determine the statistical significance between two samples, and one-way analysis of variance (ANOVA) was employed to compare the differences among three or more groups. When *p* < 0.05, the difference was deemed statistically significant.

## Results and Discussion

### Characterization of liposomes

**Figure 1:**
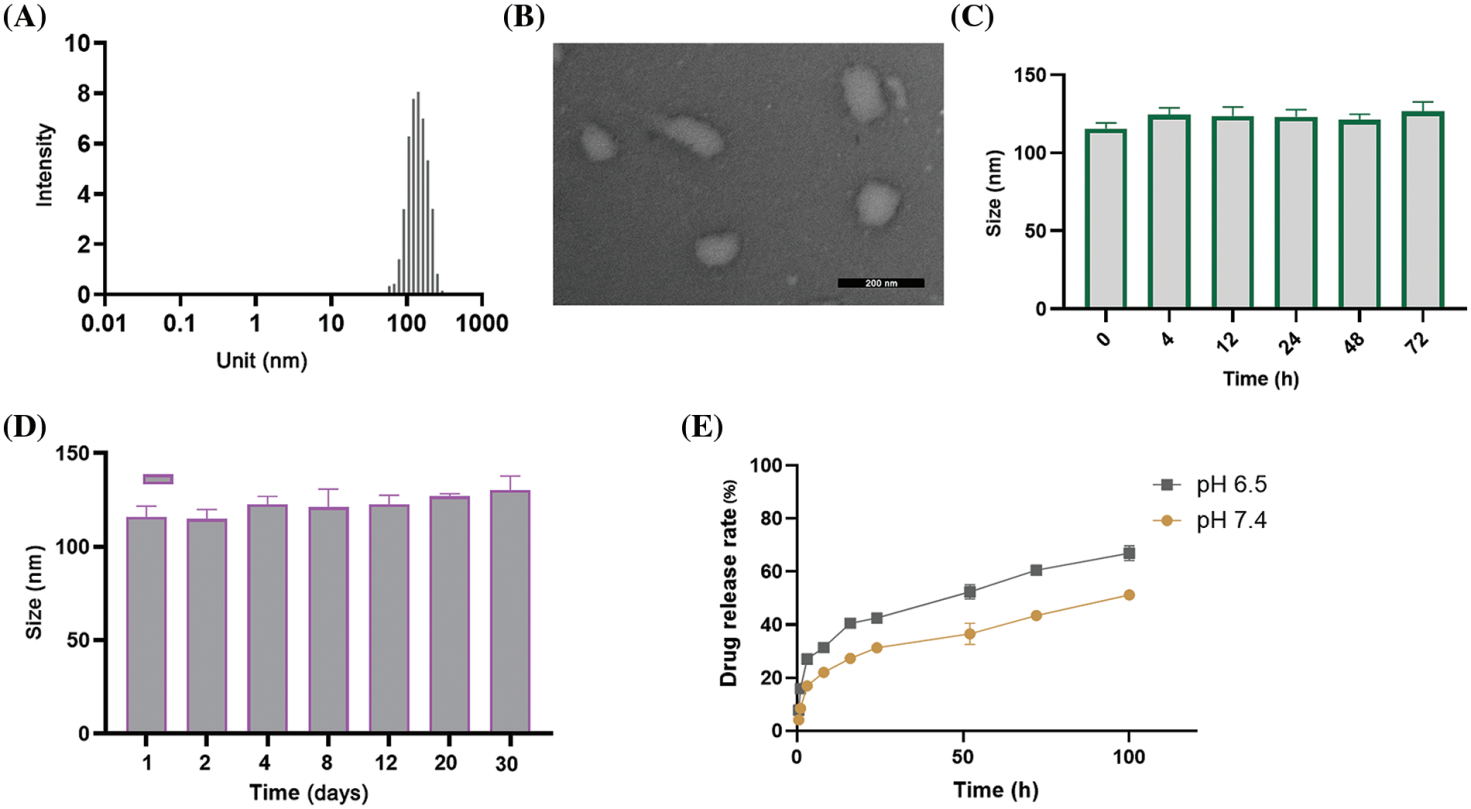
Characterizations of liposomes. (A) DLS measurement of particle size distribution of liposomes consisting of DC-CHOL, CHOL and DOPE. (B) A representative SEM image of liposomal particles. Scale bar: 200 nm. (C) Particle size changes of liposomes in PBS (pH = 7.4) containing 10% FBS after 72 h incubation at 37°C, and (D) for 30 days at 4°C. (E) The *in vitro* release profile of R848-encapsulated liposomes at various intervals in both the acidic (pH = 6.5) and neutral (pH = 7.4) environments. The data are expressed as the mean ± SD, n = 3.

As shown in Fig. 1A, the liposome sample had an average diameter of 110.1 ± 11.1 nm as determined by DLS characterization. SEM analysis of the morphology revealed a similar particle size to the DLS measurement (Fig. 1B). These observations indicate that the liposomes were successfully formed in the sample preparation. The encapsulation efficiency (EE%) of R848 in RL was 87%, and the EE% of R848 in the co-delivery liposome RTL was around 73%. These data suggest that the loading efficiency of R848 was slightly reduced by pTRAIL encapsulation (Table 1). Similarly, the encapsulation efficiency of pTRAIL was also slightly affected when R848 was loaded into the liposome simultaneously. Nevertheless, the encapsulation of these two therapeutic moieties was satisfactory in the liposomal formulation.

**Table 1:** Summary of encapsulation efficiency (EE) of R848/pDNA liposomes

Sample	Single liposomes	Codelivery liposomes (RTL)
EE of R848 (%)	87.36 ± 1.35	72.52 ± 3.11
EE of pTRAIL (%)	60.04 ± 1.05	51.1 ± 1.23

Further, the stability of the liposomes was determined by measuring liposomal particle size in PBS supplemented with 10% FBS over an extended period. The mean size of the liposomal formulations showed no significant change after 72 h incubation at 37°C (Fig. 1C) and after 30 days at 4°C (Fig. 1D). These data indicate the structural stability of the liposomal formulation. We further measured the drug release rate of the RTL liposomal formulation in both acidic (pH = 6.5) and neutral (pH = 7.4) environments. The data revealed a significant higher level of R848 drug release in the acidic environment (Fig. 1E). This is particularly relevant since tumor tissue is known to have an acidic microenvironment due to the accumulation of lactic acid [26]. Therefore, the pH-sensitive liposomes are expected to preferentially release the drug load into the TME.

**Figure 2:**
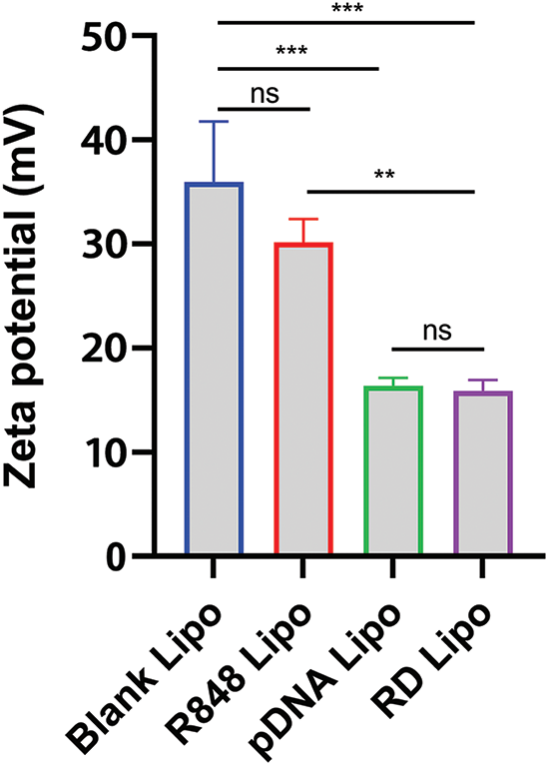
Zeta potential values (surface charge potential) were determined in blank liposomes, R848 liposomes, pTRAIL liposomes and the R848/pDNA (RTL) liposomes. Data are expressed as the mean ± SD; n = 3; **p < 0.01; ***p < 0.001.

Further, the impacts of R848 and the pDNA (pTRAIL) loading on the physical properties of R848/pDNA (RTL) complexes were determined by measuring the changes of zeta potential (surface charge potential). It was observed that both pDNA liposomes and RTL liposomes had lower zeta potentials compared to blank liposomes and R848 liposomes (Fig. 2). These data suggest that pDNA liposomes and RTL liposomes contain negatively charged plasmid DNA. The loading of pDNA in liposomes may be facilitated by the electrostatic fusion of negative pDNA with the liposomes [27].

### In vitro anti-cancer effects of liposomal formulations

**Figure 3:**
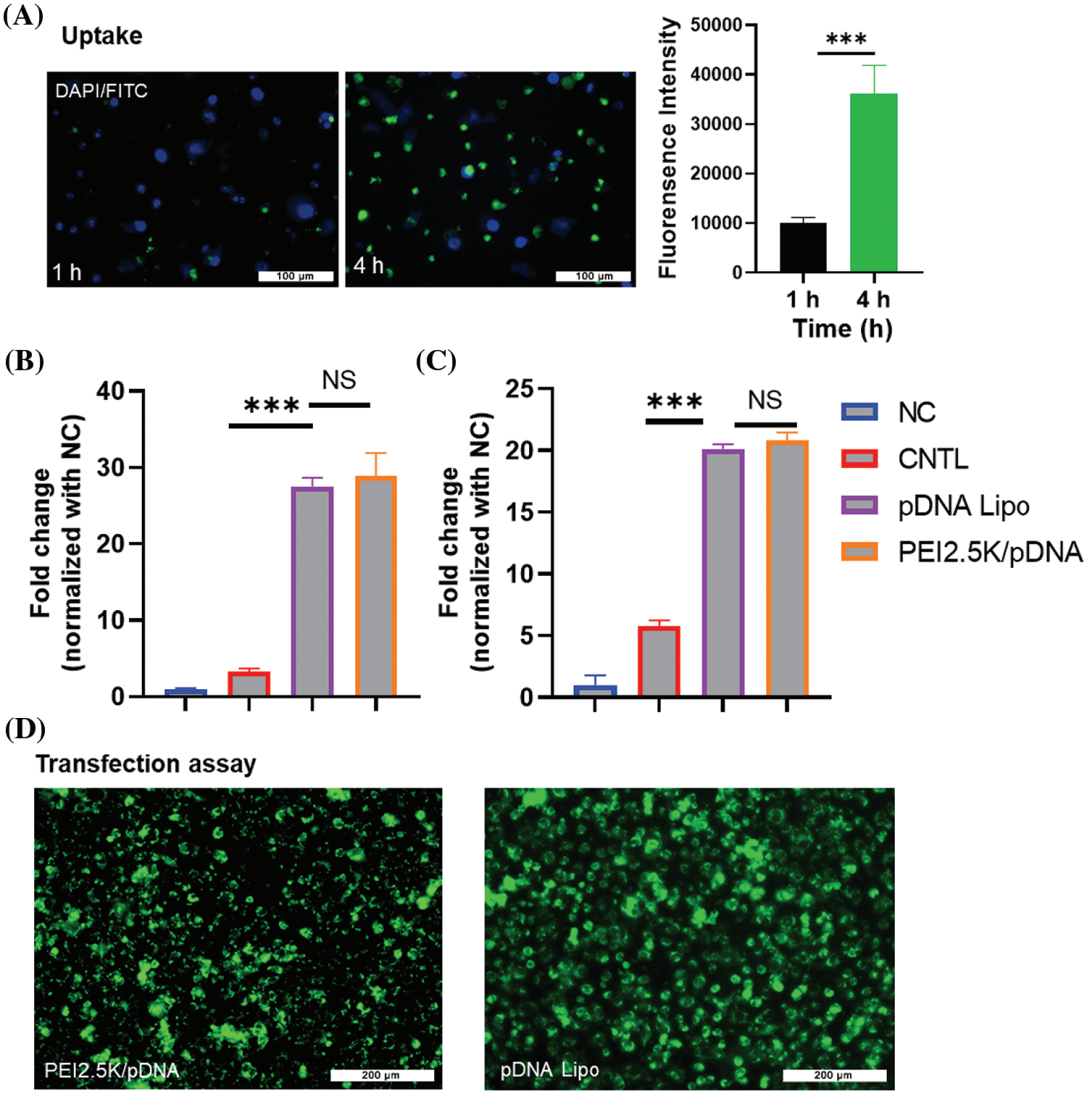
Uptake and transfection assay of cationic pDNA/liposome. (A) CT26 cells were incubated with liposomes containing GFP-expressing pDNA for 1 or 4 h. After 24 h, GFP signal fluorescence was detected. Scale bar: 100 µm. (B and C) The liposomal delivery efficiency of pDNA was compared to the PEI2.5K transfection reagent. Liposomes containing pDNA with the luciferase reporter gene were used to incubate the cells, and PEI2.5K transfection reagent was used as the positive control. The negative control (NC) represented the cells without any treatment, while CNTL represented the cells which were incubated with free pDNA only. The delivery efficiencies of pDNA in different experimental groups were measured in (B) CT26 cells and (C) HCT116 cell line based on the luciferase activity. (D) Representative images of cells incubated with pDNA liposome or pDNA/PEI2.5K transfection complex. Data are expressed as the mean ± SD; n = 3; ***p < 0.001; Scale bar: 200 µm.

Next, we prepared the liposomes containing GFP-expressing pDNA to determine the liposome uptake efficiency in CT26 cells. CT26 cells were incubated with the pDNA liposome for 1 or 4 h, and then the liposomes were removed by changing the medium. After 24 h, the detection of GFP signal in CT26 cells showed that there was a significant increase in liposome uptake after 4 h incubation when compared to 1 h incubation (Fig. 3A). Notably, most cells exhibited GFP signals after 4 h incubation, suggesting efficient internalization of the liposomes by CT26 cells. We further compared the liposomal delivery efficiency of pDNA with the PEI2.5K transfection reagent. This experiment utilized pDNA expressing the luciferase reporter gene for quantification. In both CT26 and HCT116 cell lines, the pDNA liposome demonstrated comparable efficiency in delivering plasmids to colon cancer cells as the PEI2.5K transfection reagent (Figs. 3B–3D). These data indicate that the liposomal formulation could serve as an effective vector for plasmid delivery into colon cancer cells.

**Figure 4:**
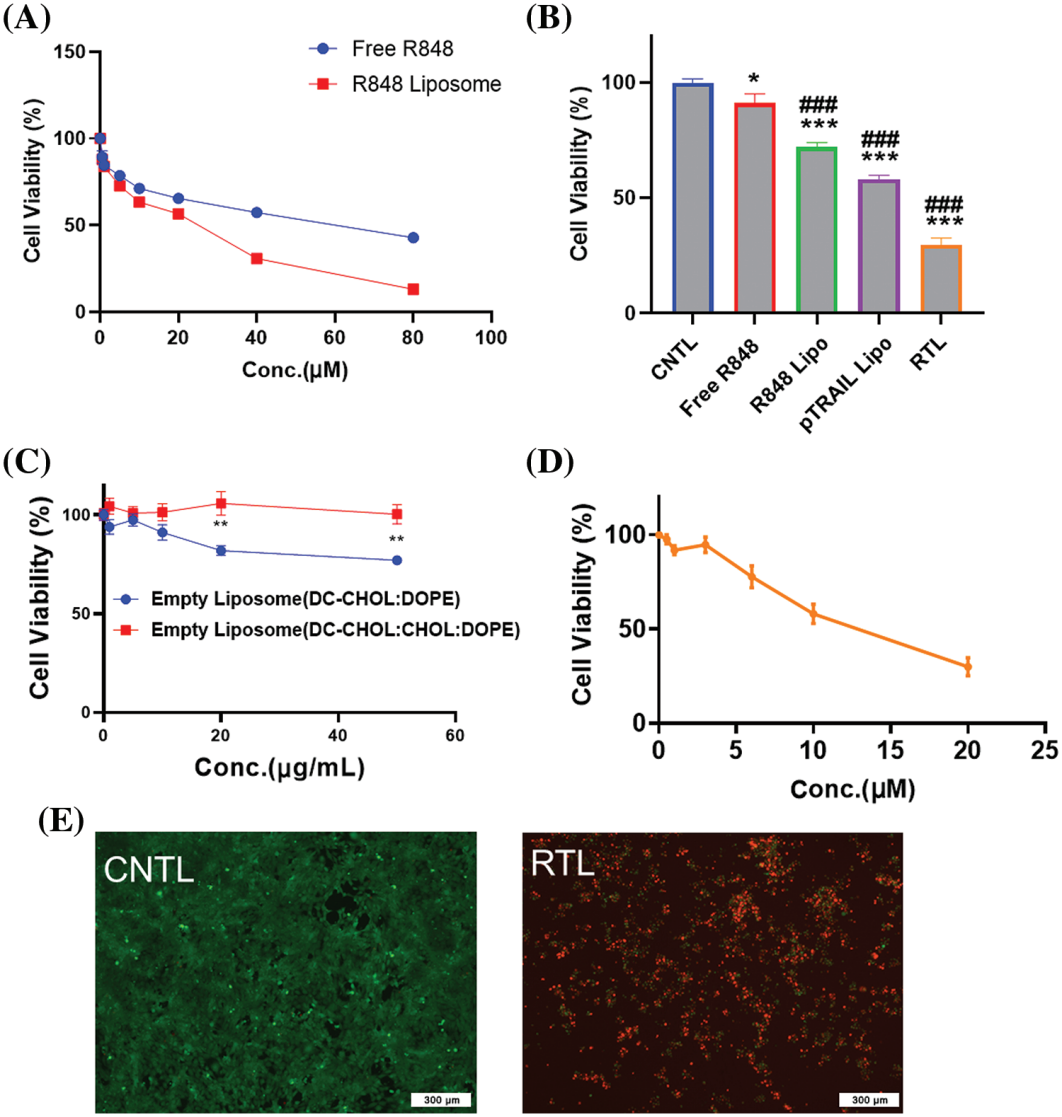
The *in vitro* anti-cancer effects of liposomal formulations. (A) HCT116 cells were treated with different concentrations of free R848 and R848 liposomes for 48 h, and a CCK8 cell viability assay was conducted to compare cell viability. (B) Cell viability analysis in HCT116 cells treated with free R848, R848-encapsulated liposome, pTRAIL-loaded liposome and pTRAIL/R848 liposome (RTL) at a concentration of R848 = 10 μM for 48 h. (C) Cell viability analysis in HCT116 cells treated with different concentrations of blank liposomes for 48 h. (D) Cell viability analysis of HCT116 cells treated with R848/pTRAIL liposomes (RTL) at different R848 concentrations (pTRAIL at 1 µg/mL). (E) Representative images of calcein AM-PI staining in control and RTL-treated HCT116 cells. Scale bar: 300 µm. Data are expressed as the mean ± SD; n = 3; *p < 0.05; **p < 0.01; ***p < 0.001 compared with CNTL group. ^###^*p* < 0.001 compared with free R848 group.

We next examined the anti-cancer effects of the liposomal formulations on the HCT116 cell line. The cells were treated with different concentrations of free R848 and R848 liposomes for 48 h, and a CCK8 cell viability assay was conducted to compare the cytotoxicity. The data showed that at the same drug concentrations, the R848 liposome exerted a much stronger toxic effect on HCT116 cells when compared to free R848 (Fig. 4A). The IC50 of R848 liposomes (22.5 μM) was lower than that of free R848 (57.2 μM), suggesting that the liposomes may be able to deliver a high level of R848 into the cells. We next compared the cytotoxicity of free R848, R848-encapsulated liposome, pTRAIL-loaded liposomes and pTRAIL/R848 liposomes (RTL) at the concentration of R848 = 10 μM. As expected, R848 liposomes showed stronger inhibition of cell viability than free R848, and the combination of R848 and pTRAIL exhibited the strongest effect on suppressing the viability of HCT116 cells (Fig. 4B). These results indicate a synergistic anticancer effect of pTRAIL and R848 in colorectal cancer cells. Of note, the presence of different concentrations of empty liposomes did not strongly impair cell viability in HCT116 cells (Fig. 4C). Moreover, the cytotoxicity effect of RTL on HCT116 cells was dependent on the concentration of encapsulated R848 (Fig. 4D). Calcein AM/PI staining confirmed that treatment with RTL significantly reduced cell viability in HCT116 cells (Fig. 4E).

### RTL regulates the expression of EMT biomarkers in HCT116 cells

Epithelial-mesenchymal transition (EMT) plays a crucial role in regulating the mobility and metastasis of cancer cells [28]. Tumor-associated macrophages are polarized into M2-like phenotype and facilitate the EMT of cancer cells [29]. Next, we attempted to examine whether RTL formulation impinges on the expression of EMT marker genes in HCT116 cells. In order to mimic the impact of tumor-associated macrophages on colon cancer cells, human monocyte THP-1 cells were polarized into M2-like macrophages and then co-cultured with HCT116 cells in a transwell chamber cassette for 48 h. The expression of EMT-related genes, including E-cadherin, N-cadherin, vimentin, Snail, Slug and Twist, was analyzed by RT-qPCR.

**Figure 5:**
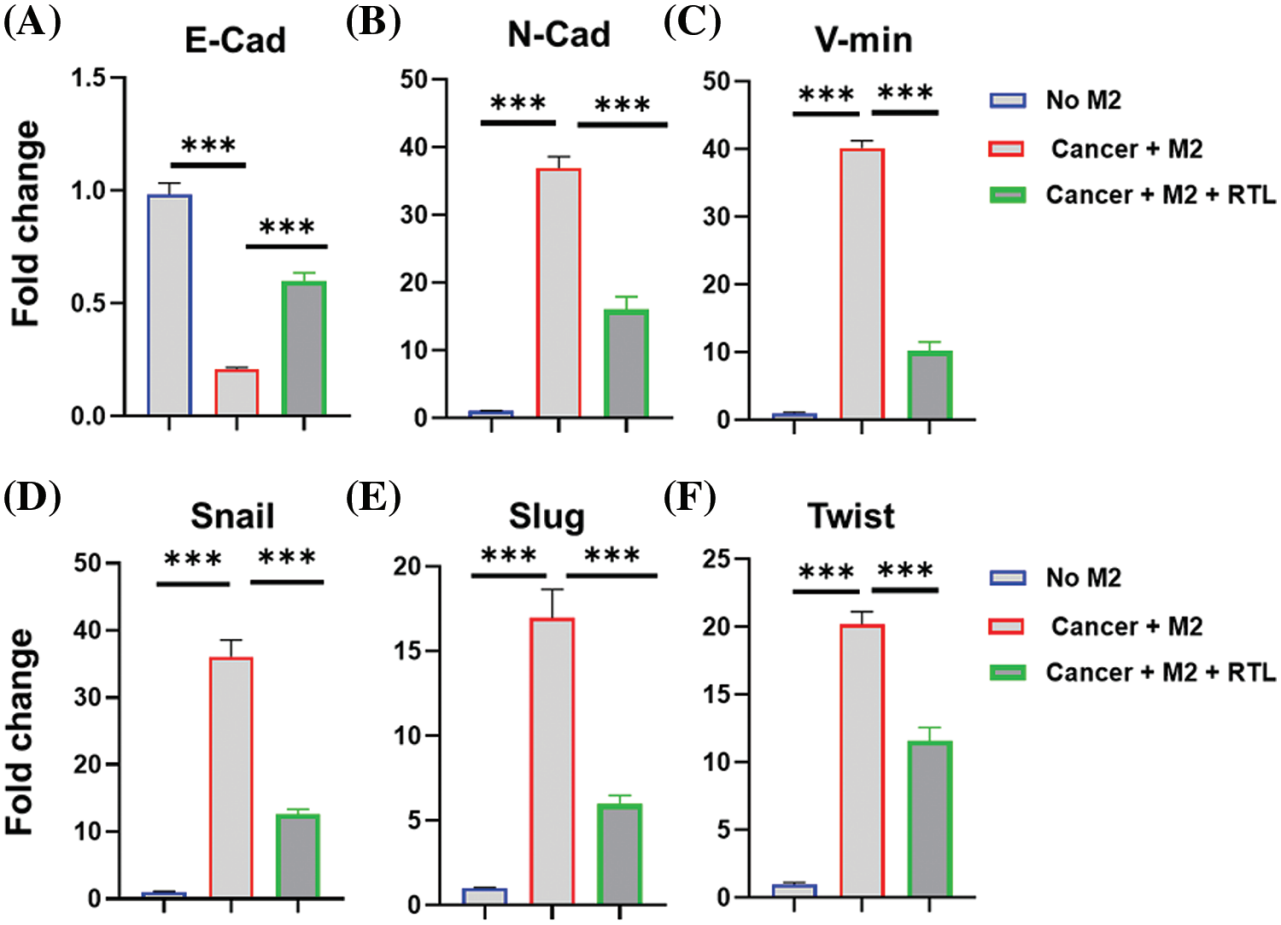
The effects of liposome treatment on the EMT biomarkers. Human monocyte THP-1 cells were polarized into M2-like macrophages and then co-cultured with HCT116 cells in the presence or absence of RTL for 48 h. qRT-PCR analysis was conducted to detect the relative expression levels of (A) E-cadherin, (B) N-cadherin, (C) Vimentin, (D) Snail, (E) Slug, and (F) Twist in HCT116 cells. Data are expressed as the mean ± SD; n = 3; ***p < 0.001.

E-cadherin is an epithelial marker which organizes the structural integrity of epithelium [30]. During the process of EMT, E-cadherin is usually down-regulated to promote cell motility and facilitate the invasion of tumor cells [31]. We observed that the co-culture with M2-polarized THP-1 cells suppressed the expression of E-cadherin in HCT116 cells, while RTL treatment increased E-cadherin level (Fig. 5A). In contrast, the mesenchymal markers, such as N-cadherin and vimentin, are up-regulated during EMT to promote cell migration [32]. In line with this, the expression levels of N-cadherin and vimentin were elevated in HCT116 cells when co-cultured with M2-polarized THP-1 cells. However, the up-regulation of this marker was significantly suppressed in the presence of RTL treatment (Figs. 5B and 5C). These data suggest that RTL has the potential to inhibit the EMT process in colon cancer cells induced by M2-like macrophages.

EMT is mediated by a group of transcription factors, including snail, slug, and twist [33]. We also observed that co-culture with M2-polarized THP-1 cells promoted the expression of these transcription factors in HCT116 cells. However, RTL treatment significantly reduced their expression in the co-culture system (Figs. 5D–5F). Since RTL treatment increased E-cadherin expression and suppressed other factors involved in the mesenchymal transition (N-cadherin, and vimentin, Snail, Slug and Twist), our data suggest that RTL formulation could inhibit M2-macrophage-induced EMT. M2-macrophage-induced EMT is a significant contributor to local tissue invasion and metastasis of cancer cells [34]. Inhibiting EMT in cancer cells has been proposed as a strategy to limit metastasis [35]. Therefore, our data indicate that RTL may also have an anti-metastatic effect on colon cancer, which warrants future investigations. Additionally, it remains to be determined whether RTL modulates the EMT status of colon cancer cells by regulating macrophage polarization.

### In vivo anticancer effect of liposomal formulations

#### In vivo antitumor efficacy

**Figure 6:**
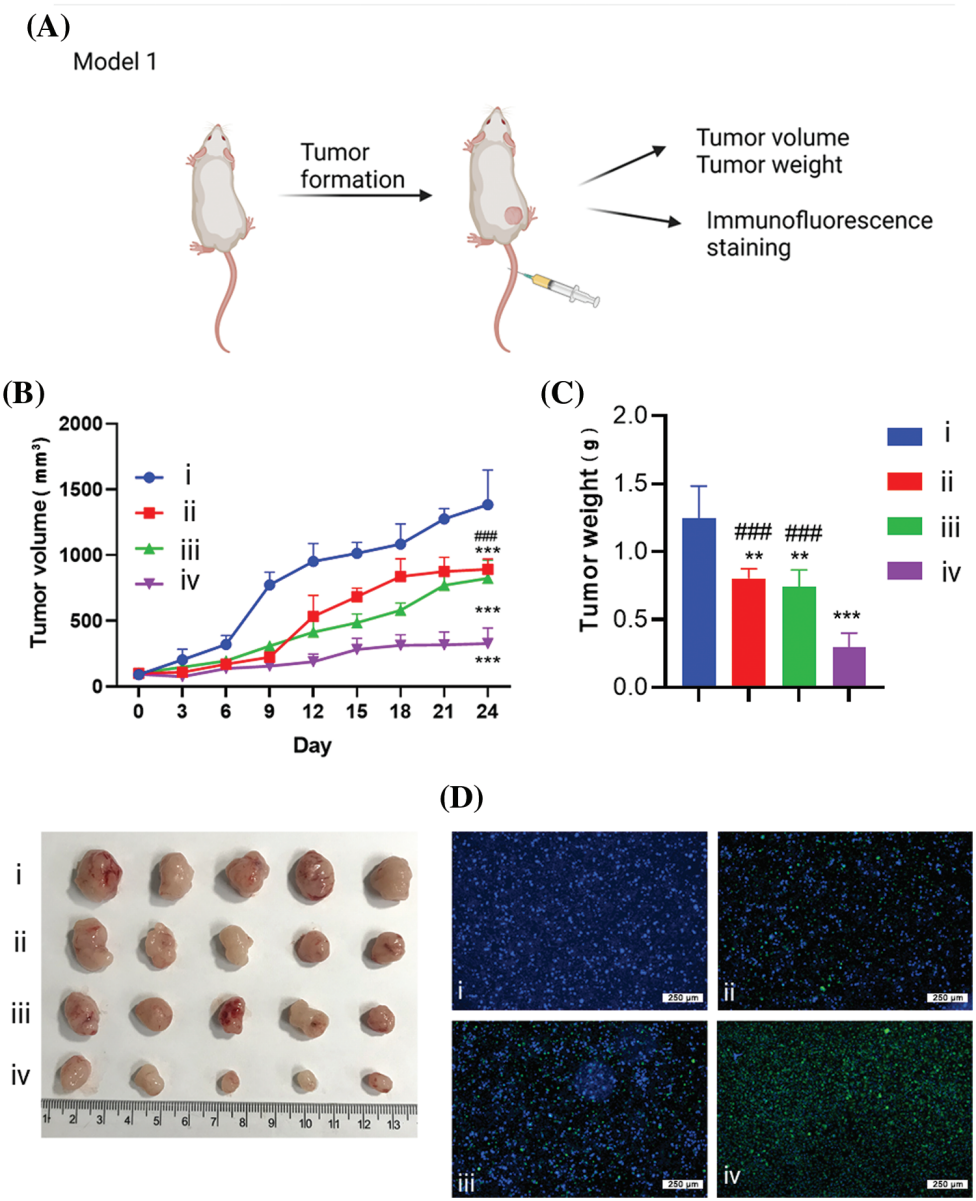
The effects of liposome treatment in a xenograft model of HCT116 cells. (A) Schematics of the xenograft tumor model establishment of HCT116 cells in nude mice. The tumor-bearing mice were randomly divided into 4 groups: (i). the control group (treated with empty liposome), (ii). the RL group (treated with R848 liposome), (iii). the pTRAIL group (treated with pTRAIL liposome), and (iv). the RTL group (treated with RTL liposome). (B) Tumor volume growth record in each group. (C) Tumor weight summary in each group. (D) TUNEL staining of apoptotic cell death in the tumor samples of each group. Scale bar: 1000 µm. Data are expressed as the mean ± SD; n = 5 animals in each group; **p < 0.001; ***p < 0.001 compared with CNTL group. ^###^*p* < 0.001 compared with RTL group.

To demonstrate the tumoricidal effect of the RTL formulation, we first established a xenograft model of HCT116 human colon cancer cells in nude mice. The tumor-bearing mice were randomly divided into four groups: the control group (treated with empty liposome), the RL group (treated with R848 liposome), the pTRAIL group (treated with pTRAIL liposome), and the RTL group (treated with RTL liposome) (Fig. 6A). The records of tumor volume and tumor weight after liposome administration showed that both RL and pTRAIL treatment suppressed the tumorigenesis of HCT116 cells in nude mice. Notably, RTL treatment exerted the strongest antitumor effect in the xenograft model (Figs. 6B and 6C). TUNEL staining in the tumor tissues further confirmed that RTL treatment caused the most abundant cell death (Fig. 6D).

#### Effect of liposomes on the immunosuppressive microenvironment

**Figure 7:**
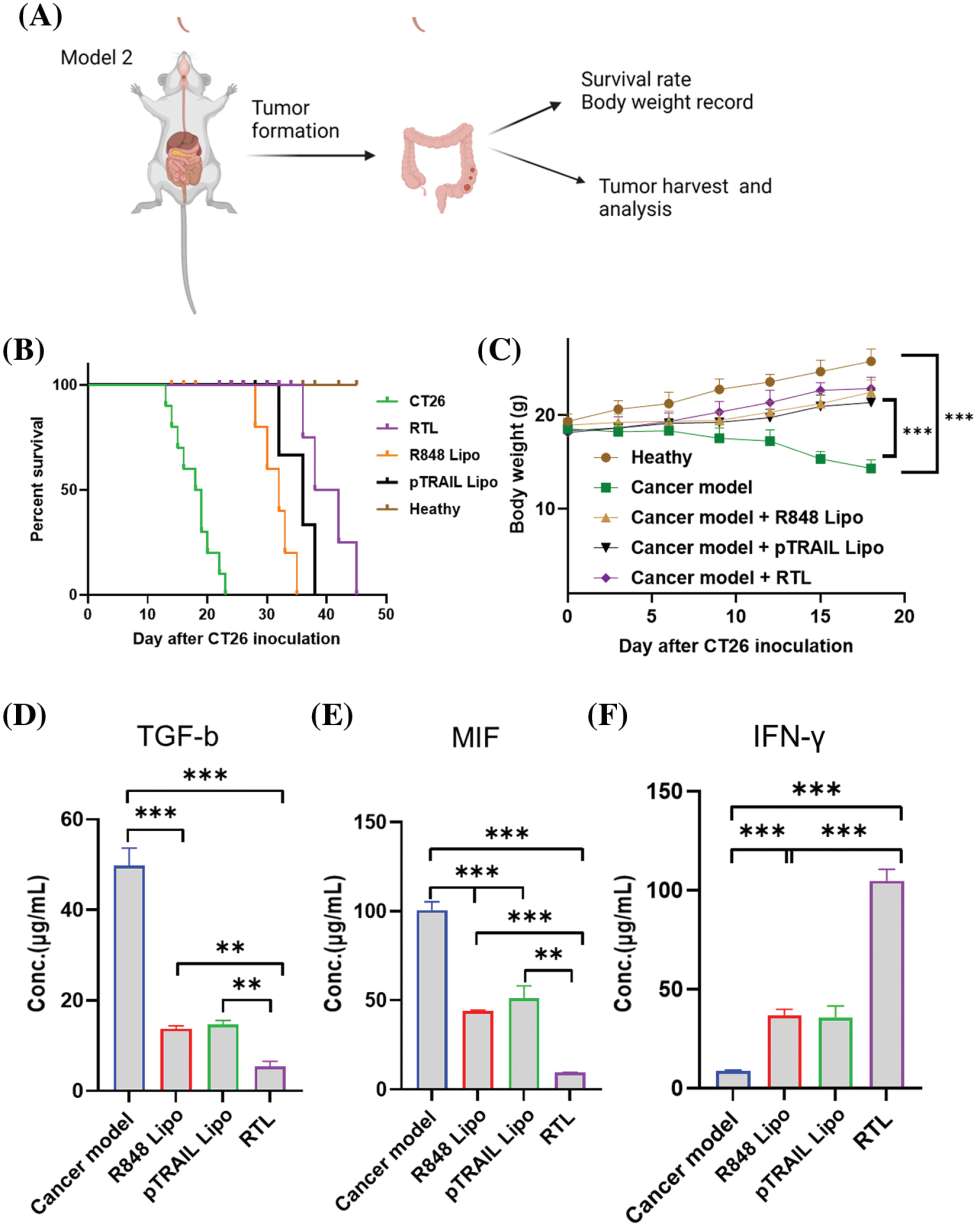
Effects of liposome treatments on cytokines in an orthotopic *in situ* model of CT26 cells. (A) The schematic illustration showing an orthotropic *in situ* model of CT16 cells at the colon tissue. The mice were divided into five groups: the healthy group (no tumor cell inoculation); the cancer model group (CT26 cell inoculation); the cancer model +R848 liposome treatment group; the cancer model+pTRAIL liposome treatment group; and the cancer mdoel+RTL treatment group. (B) The summary of animal survival in each experimental group. (C) The summery of body weight changes in each experimental group within 20 days after tumor cell inoculation. (D) TGF-β levels, (E) MIF levels, and (F) IFN-γ levels in tumor tissues of each experimental groups were detected by ELISA. Data are expressed as the mean ± SD; n = 10; **p < 0.01; ***p < 0.001.

An orthotopic *in situ* model of CT26 cells was established in the colon tissues of BALB/c mice to investigate the therapeutic effect of different liposomal formulations (Fig. 7A). In this model, the survival rate and body weight of each group were recorded. Compared to the healthy group without tumor cell inoculation, all animals injected with CT26 cells died on day 23 after tumor cell inoculation. The administration of R848 liposome, pTRAIL liposome and RTL formulation significantly extended the survival of tumor-bearing mice, with RTL treatment showing the strongest therapeutic effect (Fig. 7B). We also observed a transient decrease in body weight in tumor-bearing mice within 20 days, and this condition was improved upon the administration with R848 liposomes, pTRAIL liposomes or RTL (Fig. 7C).

In the TME, transforming growth factor β (TGF-β) acts as an immunosuppressive cytokine to promote tumor progression and confer resistance to immunotherapy. TGF-β can suppress the tumoricidal activity of immune cells and promote the expansion of the immunosuppressive cell population [36]. In the tumor tissues, we showed that treatment with R848 liposomes and pTRAIL liposomes significantly reduced TGF-β levels. The RTL formulation exhibited the strongest effect on reducing TGF-β expression (Fig. 7D). Moreover, macrophage migration inhibitory factor (MIF) is a key factor for macrophage recruitment in the TME, and tumor-associated macrophages are the key cellular components dictating the immunosuppression in the TME [37]. MIF also stimulates the production of angiogenic and growth factors to facilitate tumor progression and metastasis [38]. Our analysis showed that both R848 and pTRAIL-loaded liposomes decreased MIF levels in tumor tissues, and the RTL formulation showed an even stronger effect (Fig. 7E). On the other hand, IFN-γ is a pro-inflammatory factor that stimulates anti-tumor immunity in the TME to curb tumor development [39,40]. We found that both R848 and pTRAIL-loaded liposomes increased IFN-γ levels in tumor tissues, with the RTL formulation exhibiting the most potent effect on inducing IFN-γexpression (Fig. 7F). These findings collectively suggest that R848 or pTRAIL-loaded liposomes could reduce the expression of immunosuppressive factors (TGF-β and MIF), and promote the expression of the pro-inflammatory factor IFN-γ. The RTL formation containing both R848 and pTRRAIL exerted a synergistic effect. Taken together, our data demonstrated that the RTL formulation can potentially reactivate anti-tumor immunity by dampening immunosuppression in the TME. This effect could be attributed to the role of TLR7 agonist R848 in stimulating immune cell activity in the TME [41], as well as the immunogenicity of cancer cell death induced by pTRAIL [42].

## Discussion and Conclusion

The clinical management of colorectal cancer at an advanced stage is still challenging [43]. Targeted drug delivery and combination therapy using liposomes have emerged as a novel approach for targeting advanced cancers [44]. In the present study, we developed the RTL formulation consisting of R848 and pTRAIL as a strategy to induce cancer cell death and stimulate the immune microenvironment in colorectal cancer. We demonstrated the superior *in vitro* and *in vivo* anticancer effects of the RTL formulation compared to liposome formulations containing R848 or pTRAIL alone. In an *in situ* model of colorectal cancer, we showed that the RTL formulation extended the survival of tumor-bearing mice and could potentially stimulate anti-tumor immunity in the TME. These findings indicate the feasibility of the co-delivery of R848 and pTRAIL to achieve synergistic anticancer effects in the clinical management of colorectal cancer.

Of note, the RTL formulation also suppressed the expression of EMT markers in colorectal cancer cells that are induced by M2-like macrophages. This study has several limitations. For instance, the mechanism by which the RTL formulation inhibit cancer cell viability and stimulate the immune microenvironment in colorectal cancer remains unclear. Which immune cell populations are affected by the RTL formulation has not been investigated. Moreover, the safety of the RTL formulation needs to be evaluated before its application in pre-clinical trials. Future works are required to decipher the effects of the RTL formulation on different immune cell populations in the TME. Additionally, it would be valuable to investigate whether RTL formulation can also inhibit the metastasis of colorectal cancer in animal models.

## Data Availability

The datasets generated in the current study are available from the corresponding author on reasonable request.
